# Field Test of a Passive Infrared Camera for Measuring Trail-Based Physical Activity

**DOI:** 10.3389/fpubh.2021.584740

**Published:** 2021-03-17

**Authors:** Christiaan G. Abildso, Vaike Haas, Shay M. Daily, Thomas K. Bias

**Affiliations:** ^1^West Virginia University School of Public Health, Morgantown, WV, United States; ^2^West Virginia University Davis College of Agriculture, Natural Resources, and Design, Morgantown, WV, United States; ^3^West Virginia University Office of Health Affairs, Morgantown, WV, United States

**Keywords:** physical activity, systematic observation of behavior, passive infrared camera, field test, trail use

## Abstract

**Introduction:** Trails are ubiquitous and far-reaching, but research on the impact trails have on physical activity is limited by the lack of resource-efficient, accurate, and practical systematic observation tools. Commonly used infrared trail sensors count trail use and may broadly differentiate activity (i.e., bicyclist vs. pedestrian), but cannot detect nuances needed for outcomes research such as frequency, intensity, time, and type of activity. Motion-activated passive infrared cameras (PICs), used in ecological research and visitor management in wildlife areas, have potential applicability as a systematic observation data collection tool.

**Materials and Methods:** We conducted a 7-month field test of a PIC as a systematic observation data collection tool on a hiking trail, using photos to identify each trail user's physical activity type, age, sex, and other characteristics. We also tallied hourly trail use counts from the photos, using Bland–Altman plots, paired *t*-tests, Concordance Correlation Coefficient, Kendall's Tau-b, and a novel inter-counter reliability measure to test concordance against concurrent hourly counts from an infrared sensor.

**Results:** The field test proved informative, providing photos of 2,447 human users of the trail over 4,974 h of data collection. Nearly all of the users were walkers (94.0%) and most were male (69.2%). More of the males used the trail alone (44.8%) than did females (29.8%). Concordance was strong between instruments (*p* < 0.01), though biased (*p* < 0.01). Inter-counter reliability was 91.1% during the field study, but only 36.2% when excluding the hours with no detectable trail use on either device. Bland–Altman plots highlighted the tendency for the infrared sensor to provide higher counts, especially for the subsample of hours that had counts >0 on either device (14.0%; 694 h).

**Discussion:** The study's findings highlight the benefits of using PICs to track trail user characteristics despite the needs to further refine best practices for image coding, camera location, and settings. More widespread field use is limited by the extensive amount of time required to code photos and the need to validate the PICs as a trail use counter. The future potential of PICs as a trail-specific PA research and management tool is discussed.

## Introduction

The many physical and mental health benefits of daily physical activity (PA) are well-documented (1). Increasing population-level PA by “creating or improving places for physical activity” has for decades been recommended by the Community Preventive Services Task Force as an evidence-based community-level intervention (2). Despite the important contribution that trails may serve as public spaces for PA, “places for physical activity” has been narrowly defined in the scholarly literature as “public open spaces” such as parks or green spaces to the exclusion of trails (3, 4). In addition, Koohsari et al. (4) suggest that the correlational evidence between public open spaces and PA is mixed and due, in part, to insufficient measurement of PA in specific environmental contexts, reiterating a measurement deficiency acknowledged in the original *Guide to Community Preventive Services* (2).

Trails are vital, far-reaching places for achieving daily PA that should be considered in a more inclusive definition of public open space. Moreover, trails are a diverse and valuable community asset, accommodating many types of users and extending throughout much of the US. For instance, the trail network established by the National Trails System Act of 1968 is over 88,600 miles in length and extends to within 60 miles of 230 million residents (5). Rails-to-trails, spurred by a 1983 amendment to the National Trails System Act that allowed for the repurposing of abandoned rail-beds as multipurpose trails, now cover over 24,300 miles (6). There are also 52,600 miles of trails in the state parks system (7), and many more miles managed by city and county parks and recreation agencies.

Research of the effect of trails on *users'* PA is limited because of the lack of an efficient systematic observation method, however. Instead, most studies focus exclusively on trail *usage—*“traffic” —as the primary outcome. Methods most often used to collect trail usage include: (a) infrared sensor counters (8), (b) survey self-report of nearby residents (9–13), and (c) in-person systematic observations (14). Like vehicular traffic counts, trail usage counts are used to estimate annual volume of traffic on trails based on a sample of daily counts, resulting in an estimated average annual daily traffic (AADT) on a trail (15, 16). While requiring few resources, infrared sensors are limited in the output they produce to counts or “uses,” systematically undercount groups of users as a single “use,” may mistakenly count animals, and—importantly for public health researchers—cannot identify trail user characteristics or PA type (15, 17, 18).

Trail usage counts lack the specificity needed to assess and quantify PA frequency, intensity, time, and type (FITT) (19) of *each trail use and user*. Systematic observation is generally considered the “gold standard” for collecting place-based PA data in public open spaces. Applied conceptually to the context of a trail, a systematic observation would allow for the identification of specific *trail users* so that the PA type of each user could be assessed at an observation location (i.e., trail access point), in addition to the quantification of *total trail use*, or traffic counts. A systematic observation can also be used to quantify each user's characteristics (e.g., age, sex, group usage, helmet use) for research and management purposes. However, in-person systematic observation studies require substantial human and financial resources (18). For example, systematic observations require a long data collection period—or a sample with extrapolation to a longer period to estimate AADT. Using in-person observation to assess the PA time and intensity of a trail user further requires the capability to identify and track specific individual users while on the trail, exacerbating the resource needs and making such an observation untenable, particularly on longer distance trails and/or trails with numerous access points.

Because of the resources required to conduct in-person systematic observations, most trail-based PA studies to date have used cross-sectional surveys to capture samples of residents living near a trail (9, 11, 13, 20) or individuals intercepted while using the trail (21–23). Surveys are beneficial for gathering trail-based PA data that are needed to quantify individual-level impacts such as sociodemographic information of users and details of the PA FITT. However, cross-sectional surveys are often subject to recall, selection, and temporal biases. Moreover, implementing in-person surveys often requires more time and coordination to ensure fidelity to research protocols which may lead to higher costs. In an attempt to bridge research gaps, studies have combined trail use sensor count data with trail user intercept survey responses to estimate population-level impacts. However, these methods rely heavily on assumptions about frequency of trail use to calculate user-specific PA (24). These efforts highlight the need to identify and test a resource-efficient solution that can be used over an extended period of time to simultaneously quantify trail *use* and each individual *user's* trail-specific PA FITT.

An accurate assessment of PA type, time, and intensity of each trail user requires the ability to observe each user's entry and exit from the trail. For example, a 24-h systematic observation at every access point of a trail could be used to track the time each individual user spends on a trail and the PA type and intensity at each observation point. Additionally, assessing PA *frequency* requires repeating this observation over multiple days or weeks. Thus, an accurate assessment of all dimensions of FITT requires the ability to track each uniquely identified trail user at each trail use and determine if each trail user returns, but raises scalability, ethical, and data processing concerns as noted in the literature (25). Even if accomplished, assumptions that each user's time on the trail was spent on one PA type and at a consistent intensity would still need to be made. Conducting an in-person systematic observation at every minute of every day over multiple weeks would further intensify the resource needs, but it may be possible with technological advances.

Remote cameras have been used as a systematic observation data collection tool in ecological research and visitor management in wildlife areas to: (1) quantify animal and human visitors and/or interactions (26–28), (2) protect wildlife (29, 30), and (3) monitor for park policy violations (31). These passive infrared cameras (PICs)—also known as wildlife cameras, game cameras, or camera traps—take photos and/or videos when activated by motion or a heat source, providing details in each photo that could be used to detect individual users' PA type (26, 27). While PICs eliminate the need for humans to conduct in-person counts, coding of images or videos to glean information is still required. Only recently, however, have researchers attempted to demonstrate the applicability of using the photos from PICs to quantify PA of human users of trails (27, 32). Developing best practices has been difficult because there have been very few studies, protocols vary, there are numerous PIC brands and models, and PIC technology and device features are constantly improving. Despite such limitations, PICs may be an efficient and practical data collection tool for systematic observation over long periods at multiple sites on a trail. A key initial step is to field test PICs to determine the feasibility and dependability of PICs as a data collection tool for assessing PA type of trail users. Thus, the purpose of this study was to field test a PIC on a hiking trail and evaluate its use as a tool for identifying the PA type and characteristics of individual trail *users*. A secondary purpose of this study was to field test a PIC as a trail *usage* counter by comparing it with concurrent counts from an infrared sensor.

## Materials and Equipment

### Instruments

Data for this study were collected using a PIC concurrently with an infrared trail count sensor at the main entrance to a hiking trail in a newly established urban woodland park. The protocol was reviewed by the West Virginia University Institutional Review Board (protocol number 1905572292) and deemed Non-Human Subjects Research because it was an observation of public behavior.

#### Passive Infrared Camera

A Moultrie M-888i Mini Game Camera, mounted in a camouflaged protective case (CAMLOCKbox) and secured with adjustable (Python) cable lock, was tested. The M-888i triggers with motion or heat within a 15-m range using a passive infrared sensor. It has the capability to capture photos or videos. For this study, it was used to take photos. It captures night images by using an infrared flash. This M-888i uses eight AA batteries, intended to capture and store up to 17,000 images on a removable SD-card (33). This camera is no longer manufactured (retail $126) but has been replaced by the M-40i model (retail $160). The cost of the case, cable lock, SD-card and initial set of batteries added $78 to each camera setup.

For this study, important camera settings included: (1) detection delay and (2) multi-shot option. Detection delay refers to the amount of time between pictures when an object is detected and remains in range. Options are 0, 10, or 30 s, or 1, 5, 10, or 30 min. Multi-shot refers to how many pictures are captured once the camera is triggered. Options are: (1) “off” for a single photo per triggered event; (2) “burst” for three photos within 1 s per triggered event; or (3) “triggered” for up to three photos, with a slight delay between each (if the subject continues to move in view of the camera after the first photo triggering event). To protect anonymity of trail users photographed in a public space, faces were obscured in images selected as illustrations, and we only selected photos of trail users from whom we had a media release.

#### Infrared Trail Count Sensor

For our comparison, we used a TRAFx infrared trail counter, which detects the “infrared wavelength that people emit” (34). The sensor uses an infrared scope connected to hardware inside a weatherproof case. The unit runs on three AA batteries, with an estimated battery life of up to 3 years. Settings for the infrared counter include the: (1) Period and (2) Delay. Period allows the user to choose between timestamps for each triggering event (“sensor hit”) or a summary count of sensor hits per hour or per calendar day. The Delay setting establishes the minimum time between each triggering event in 0.25-s increments. When purchased in 2015, this counter and weatherproof case cost $520. A dock to download the data cost an additional $550 and optional online software to view and manage the data can be added for approximately $100 per year. Batteries and a steel electrical disconnect box ($15) in which to lock the equipment cost extra.

## Methods

### Data Collection

**Figure 1:**
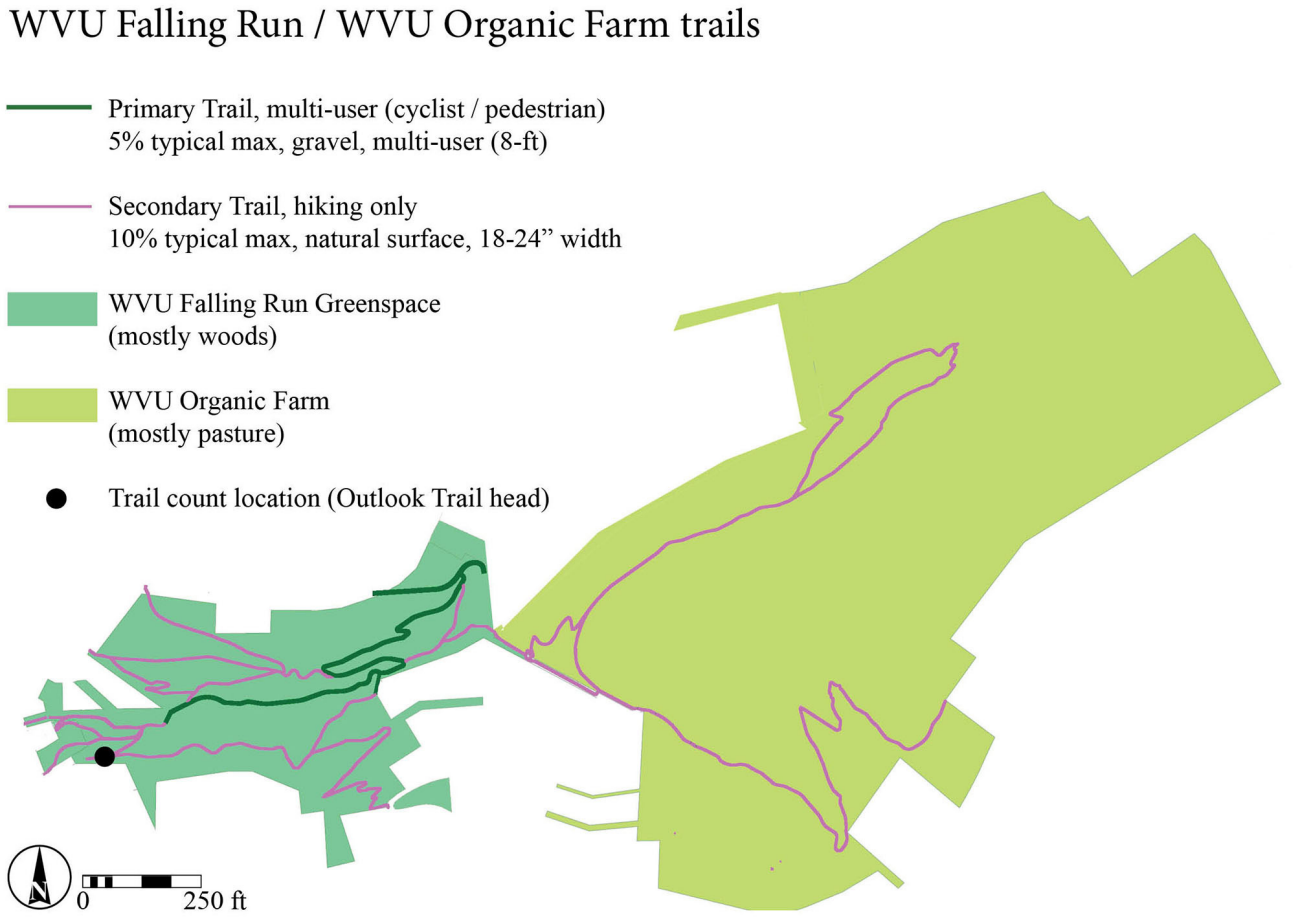
Map of the Falling Run Greenspace/WVU Organic Farm, study site and trail count location in Morgantown, West Virginia, USA.

**Figure 2:**
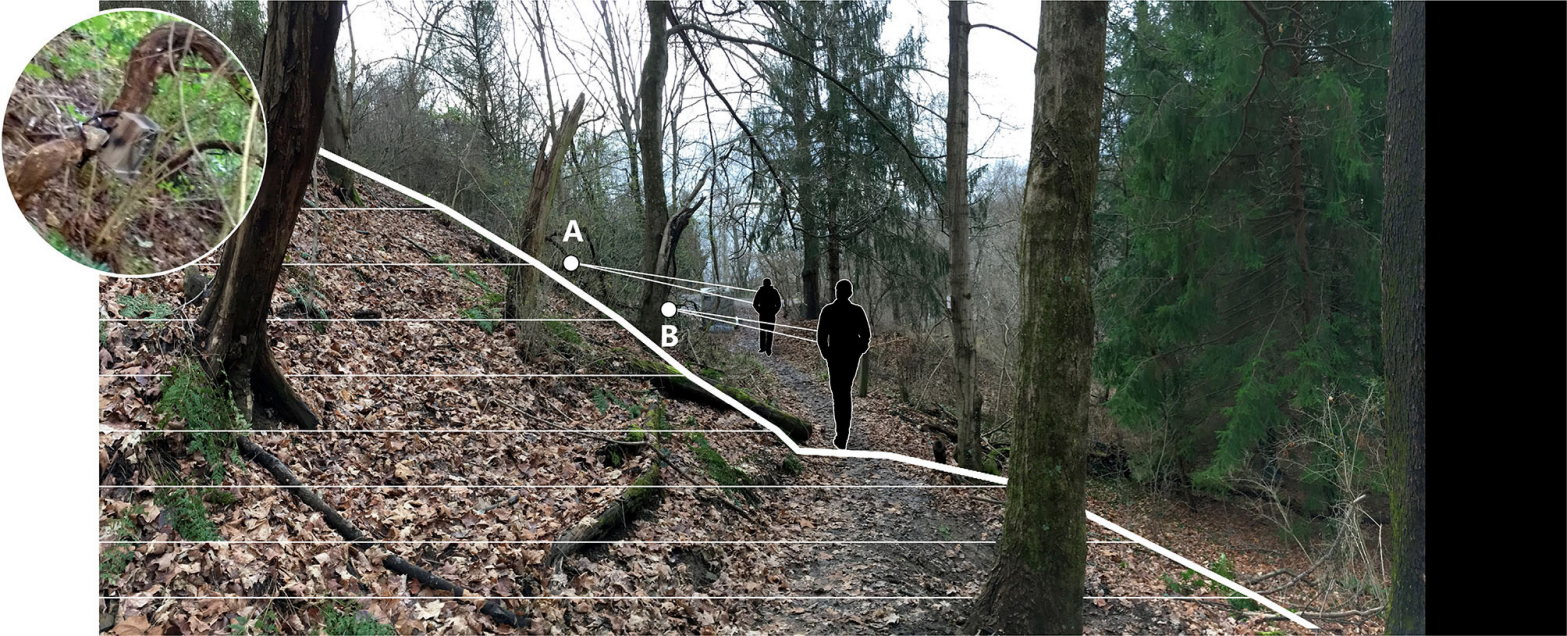
Image depicting the field test location, looking toward the trail entrance, with the location of the PIC **(A)** and infrared trail sensor **(B)** noted. The PIC was 3 m from the trail edge and the sensor was 1 m from the trail edge. The inset photo is an image of the PIC mounted to sturdy grape vine at location **(A)**. PIC, passive infrared camera. Bold line shows cross section of topography at infrared sensor location (foreground). Horizontal lines denote 0.6-m contours.

Data were collected from November 19, 2016 to June 30, 2017 at the main entrance of a hiking trail accessing the West Virginia University (WVU) Falling Run Greenspace/Organic Farm, a 60-acre wooded park in Morgantown, West Virginia, USA (Figure 1). This trail is designed for foot traffic only; bicycles are not allowed but were observed occasionally in the present study. Preliminary instrument set up and mounting angle tests were conducted prior to full data collection to ascertain that photos captured bi-directional walking and jogging along the trail concurrently with infrared sensor timestamps. The PIC was cable-locked within a metal security box, mounted to a 10-cm diameter grapevine 3 m from the edge of a 0.6-m wide hiking trail. The camera had an unobstructed view perpendicular to the trail from the uphill slope at roughly 1.8 m above the trail surface. The camera was angled downward to parallel the steep hillslope (59%) to capture trail users' full-body lengths (Figure 2). These specifications are slightly different than the recommendations suggested by Miller et al. (32), but were found to most accurately capture walkers and runners of different heights and different speeds during preliminary tests, accounting for the width of the trail and topography, where prevailing hillslopes are often 30% or more. The camera was set to 10-s delay with a “burst” of three exposures per triggering event. Images contained a date and timestamp, and were downloaded to a password-protected laptop from the removable SD card monthly. Batteries needed to be replaced roughly every 2–4 weeks.

The TRAFx infrared sensor was mounted to a tree in a locked metal box about 1 m from the trail's edge with an unobstructed view (Figure 2). The Delay option was set at 1.5 s and the Period option was set to provide hourly trail usage counts. Hourly trail usage counts were downloaded monthly through a wired connection to a password-protected laptop as text files and converted to a Microsoft Excel spreadsheet. Batteries did not need to be replaced during our data collection period. The date and time were synchronized on the PIC and infrared sensor at initial setup, and verified during each visit to the instruments to download data.

### Analysis

The second author reviewed all photos from the PIC to code the characteristics of each user. Codes included: (1) the type of PA being performed (running, walking, bicycling) based on body lean, stride length, and equipment (NB: bicycling not permitted, but was detected); (2) biological sex (male, female) based on cultural cues such as dress and hair style, and/or by visible physical features such as facial hair or body proportions; (3) age group (child or adult) based on individual height and features and accompanying caregivers; (4) dog walking (yes, no); (5) mobile phone use, either viewing the screen or holding it up to the ear; and (6) date and time of day from the timestamp on the photo. Group size was coded by counting the number of unique trail users photographed during the multiple photos for each triggered event. Photos triggered by animals (unaccompanied pets, wildlife) were coded, but excluded from our analyses. When some distinguishing item of clothing, a distinctive pet, or other features made it possible to recognize a unique human trail user, the duration of that persons' visit was noted and incidents of their repeat trail use were noted as was feasible. The date and time from the timestamp of each photo were used for aggregation into hourly trail user counts for comparison with the hourly counts from the infrared sensor data.

Descriptive analyses included frequencies with valid percentages for categorical or count indicators and measures of central tendency with variance for continuous indicators. To explore the agreement or consistency (i.e., concordance) between the hourly counts from the infrared sensor and hourly user counts derived from the PIC photos, this study followed methods outlined by Johnson and Waller (35) and similar to those used in studies of systematic observation methodologies of PA in park spaces (36, 37). Specifically, Bland–Altman plots (38), paired *t*-test, Concordance Correlation Coefficient (CCC) (39, 40), and Kendall's Tau-b (41) were used to test for agreement and bias between instruments. Bland–Altman plots are a visual tool used to evaluate bias between the mean differences of the infrared sensor and PIC hourly counts (42). CCC interpretations are: small (≤0.40), moderate (0.41–0.60), large (0.61–0.80), and very large (0.81–1.0) (36, 43). Kendall's Tau-b ranges from −1 to +1, or from perfect negative to perfect positive relationship. Measures were checked for non-normality and logarithmically transformed where appropriate based on guidance from Giavarina (42). Kendall's Tau-b was selected as a non-parametric alternative for comparisons as recommended by Johnson and Waller (35). Statistical significance was determined with an alpha-level set to 0.05 using a two-tailed distribution. Sensitivity analyses were performed to compare statistical inferences between the full sample of hourly counts and those hours when the count on at least one device was not zero. All statistical analyses were performed using SAS 9.4® (44).

In addition, an inter-counter reliability (ICR) was used to calculate the percent agreement between the hourly user counts derived from coding of PIC photos and infrared sensor hourly counts. This method, albeit exploratory, was chosen as an equivalent to interrater reliability measurement that is used to assess agreement for observational measures in qualitative research (45). To calculate ICR, hourly counts were first matched by date and hour from each device and coded as either *agreement* (sensor count = PIC count) or *disagreement* (sensor count ≠ PIC count) for each hour. Then, the percent of hourly trail use counts in agreement (number of hourly agreements/[number of hourly agreements + number of hourly disagreements]) was calculated. An agreement of 80% or higher was considered “acceptable” using the ICR method based on standards from content analysis and other qualitative coding methodologies (46–49).

## Results

**Figure 3:**
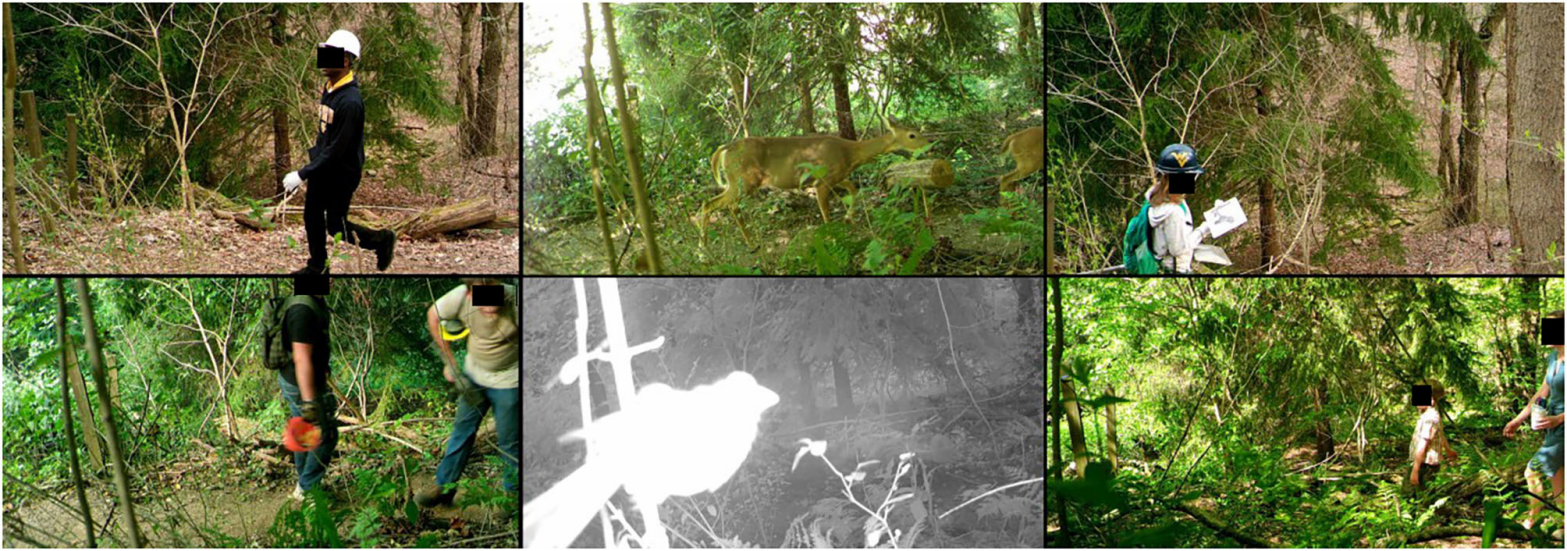
Example images from passive infrared camera field test.

In sum, 4,974 h of matched data from both instruments were collected during the field test, excluding an interval when the PIC's batteries expired (384 h from January 26 to February 11, 2017). Coding the PIC images (see examples, Figure 3) took roughly 30 s per triggering event. The camera also captured many photos with no discernible trail user, many of which we attributed to wildlife, wind-induced movement in background vegetation, and insects triggering the camera at close range. Some unknown triggers could have been due to high-speed users, such as runners or cyclists.

Trail user PA and demographic characteristics derived from the photos are presented in Table 1. The PIC recorded 2,447 trail users and 253 animals. Nearly all of the 2,447 human users were walkers (94.0%), 3.6% were children, 2.4% were using a mobile phone, and 59.6% were in a group of two or more. The majority of trail users were male (69.2%). Females had a lower rate of solo trail use (29.8%) than did males (44.8%).

**Table 1:** Trail user characteristics based on coding of images from a PIC on a hiking trail.

	**Total (*****n*** **= 2,447)**		**Male (*****n*** **= 1,600)**		**Female (*****n*** **= 711)**	
**Sample characteristic**	***n***	**%**	***n***	**%**	***n***	**%**
**Activity type**						
Walking	2,299	94.0	1,512	94.5	677	95.2
Running	116	4.7	78	4.9	35	4.9
Bicycling	31	1.3	18	1.1	5	0.7
**Trail users**						
Groups	606		^*^214		^*^62	
Users in groups	1,457	59.5	482	30.1	133	18.7
Solo	989	40.4	717	44.8	212	29.8
**Walkers**						
Groups	578		^*^205		^*^57	
Users in groups	1,391	56.8	464	29.0	123	17.3
Solo	908	37.1	660	41.3	199	28.0
**Runners**						
Groups	17		^*^7		^*^5	
Users in groups	36	1.5	14	0.9	10	1.4
Solo	68	2.8	50	3.1	15	2.1
**Bicyclists**						
Groups	4		–		–	
Users in groups	8	0.3	^*^n/a		^*^n/a	
Solo	21	0.9	14	0.6	3	0.1

*PIC, passive infrared camera*;

* *unique number of groups composed exclusively of a single biological sex*.

Comparison statistics between infrared sensor and PIC are available in Table 2. Counts demonstrated a substantial number of hours matched with zero counts on both devices (86.1%). With zero count hours included, the infrared sensors recorded a summary count of 3,022 and a mean of 0.6 (SD = 4.4) per hour. The PIC recorded a sum of 2,447 users and a mean of 0.5 (SD = 2.9) trail users per hour. The test for minimal bias using a paired *t*-test was significant (*t*_4973_= 3.64, *p* < 0.01), revealing bias between the counts. The concordance test showed very large agreement between counts (*r*_c_ = 0.93, 95% CI: 0.92–0.94, *p* < 0.01). Kendall's Tau-b demonstrated a significant relationship between counts (τ_b_= 0.89, *p* < 0.01). The inter-counter reliability with zeros included was 91.1% (ICR = 0.91).

**Table 2:** Comparison of hourly count data from passive infrared camera and infrared sensor on a hiking trail.

	**Full sample (*****n*** **= 4974)**				**Hours with counts >0 on at least one device (*****n*** **= 694)**			
	**IS**	**PIC**	**Diff**	**Mean**	**IS**	**PIC**	**Diff**	**Mean**
Count, sum	3,022	2,447	575	2,735	3,022	2,447	575	2,735
Count, per hour, *M* (SD)	0.6 (4.4)	0.5 (2.9)	0.1 (1.9)	0.5 (3.6)	4.4 (11.1)	3.5 (6.9)	0.8 (5.0)	3.9 (8.9)
Hours with 0 count, *n* (%)	4,354 (88%)	4,315 (87%)			74 (11%)	35 (5%)		
*t* (df)	3.64 (4973)^*^				3.67 (693)^*^			
*r*_c_ (95% CI)	0.93^*^	(0.92, 0.94)	0.08^*^		0.77^*^	(0.74, 0.80)	0.02	
τ_b_	0.89^*^				0.60^*^			
ICR	0.91				0.36			

* *p < 0.05*.

*PIC, passive infrared camera; IS, infrared sensor; Sum, summation of hours, M, sample mean of device counts; SD, standard deviation of device counts; t, paired t-test; r_c_, concordance correlation; τ_b_, Kendall's Tau-b; ICR, inter-counter reliability; Diff, (IS—PIC); Mean = (IS + PIC)/2*.

**Figure 4:**
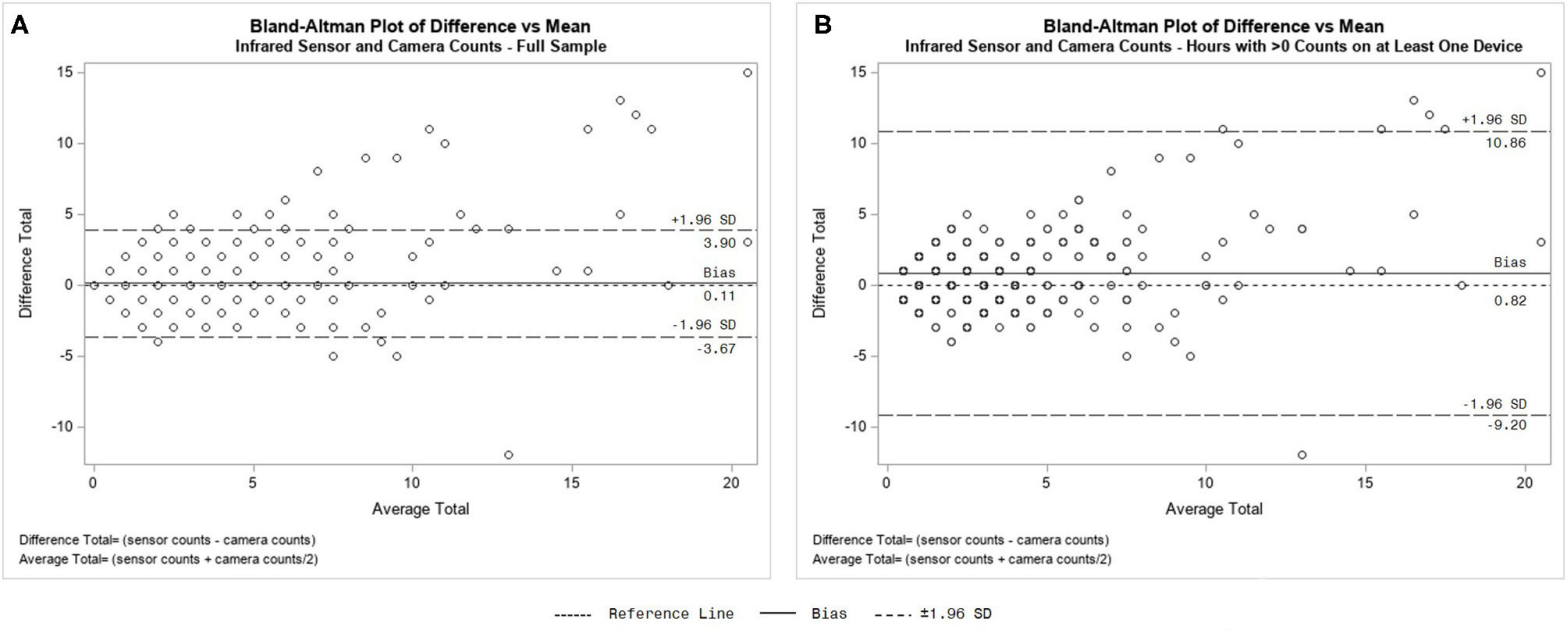
Bland–Altman plot of infrared sensor hourly counts and hourly counts derived from passive infrared camera photos for full sample of hours **(A)** and the hours with count > 0 on at least one device **(B)**.

For the subset of hours that excluded the hours with matched zero counts (*n* = 694) the infrared sensors recorded a summary count of 3,022 and a mean of 4.4 (SD = 11.1) per hour. The PIC recorded a sum of 2,447 users and a mean of 3.5 (SD = 6.9) users per hour. The test for minimal bias using a paired *t*-test was significant (*t*_693_ = 3.67, *p* < 0.01), revealing bias between the counts. The concordance test showed large agreement between counts (*r*_c_ = 0.77, 95%; CI: 0.74–0.80, *p* < 0.01). Kendall's Tau-b demonstrated a significant relationship between counts (τ_b_ = 0.60, *p* < 0.01). The inter-counter reliability for this subset of hours with zero counts excluded was 36.2% (ICR = 0.36). Lastly, the Bland–Altman plots (Figure 4) show the error and bias, outliers, and difference trends relative to the mean for the full sample and for the subsample of hours with non-zero counts on at least one device. These plots visually represent the finding that the infrared sensor had higher counts for 34.9% of the hours of non-zero counts.

## Discussion

Among the research needs for public open spaces, Koohsari et al. (4) identified the need to establish “open space-specific measures of physical activity” (p. 80). Based on an extensive review of the literature, this appears to be the second field test of PICs as a systematic observation data collection tool for trail-specific PA (32). We were able to collect 4,974 h of concurrent data with one period of missing data due to battery expiration in the PIC, highlighting a logistical concern to consider in the field. Newer versions of the camera can be connected to a cellular network for a monthly fee that also allows users to use a smartphone application to get low battery, low storage space, and other warning notifications. Regardless, peripheral expenses such as batteries and storage space should be accounted for when budgeting. Photos provided detailed information about trail users and allowed for the removal of animals from counts, so that we were able to discern that the vast majority of trail users were walkers and males more often used the trail alone than females. These advantages over hourly usage counts from infrared sensors came at a cost—the time required and potential bias of coding images by hand—that should be addressed in future studies.

Our findings highlight the benefits of using PICs to track trail *user* characteristics despite the need to further refine best practices for camera location and image coding. For example, Miller et al. (32) recommended that cameras be placed 1–2 m from the trail edge at a 20° angle to the trail, and at knee-height (0.5 m) to capture the average mountain biker between 8 and 16 kph. During the preliminary setup, we made the decision to place the PIC farther from the trail, at a right angle to the trail, and at a higher location than Miller et al. (32) recommendation for three reasons: (1) the steeply sloping topography of the study location, (2) the trail in this study is not intended for use by people on bicycles, and (3) we wanted to capture as many identifying characteristics of the trail users as possible. For future PIC installations, we recommend multiple and extended periods of in-person testing at the site to identify the optimal mounting location and camera settings to capture a wide range of trail user speeds.

This study's field test demonstrated the potential of PICs as a trail-specific PA research tool. Discernable from the photos were: (1) PA type using the lean, stride length, and visible equipment (e.g., bicycle); (2) the directionality of users; (3) demographic characteristics for most users; and (4) diverse purposes of trail use based on visible equipment (e.g., cameras, hula hoops, juggling pins). As tested, this single-camera setup is useful for identifying trail usage by type, sex, time of day, and day of week, and whether dog walking, group usage, and/or disallowed uses (i.e., bicycling) are occurring *at a single location*. With methodological creativity and technological innovation, PICs could be used in a long-term, multi-location setup to estimate: (1) the number and characteristics of each distinct trail user; (2) the PA intensity and time of each user while on the trail; (3) the PA type and trip type of each user (round-trip or one-way); and/or (4) demographic differences in PA FITT and use preferences. For example, with cameras located at every entry point of a trail system, it becomes possible to track each user and estimate PA FITT in that setting by using unique features (e.g., facial features, clothing) to distinguish individual trail users. Identifying specific people and coding images will require technological innovation, such as using computer vision algorithms (36, 37). By achieving this capability, researchers could answer Koohsari et al. (4) call for evidence of causal inferences. That is, researchers could track setting-specific PA over a period of time when trail improvements occur such as building additional trailheads or installing lighting, during a specific event on a trail (e.g., National Trails Day), or as a result of a specific intervention that encourages trail use among a demographic group (e.g., children, females, people of color).

Despite limitations in coding based on superficial characteristics in photos that may introduce coder bias, such as age and sex, our field test also demonstrated the potential that PICs have for trail and park management through the identification of particular user and group characteristics including trail use preferences. Future studies of inter-coder reliability are necessary to overcome coder bias and help develop image recognition capabilities for fast, accurate coding using emerging technologies. While demographic characteristics such as age and sex of users were not always discernable from the photos, we observed in the codable images that the majority of users were male, and females tended to use the trail more often in groups. Such details about user characteristics, possibly in combination with trail user survey data, could help land managers identify programming or infrastructure improvements to promote inclusivity for users.

There are three important barriers to overcome to make it feasible to use PICs as a systematic observation data collection tool to quantify trail-based PA and trail usage. First, is the time required to do the manual coding of the images. This could be informed by the evolution that has occurred in the systematic observation instrumentation of PA in public spaces and parks/recreation areas. The methods for assessing pedestrian and bicyclist usage of public spaces such as plazas and on-street transportation infrastructure could be particularly informative. Through progressive studies using the Archive of Many Outdoor Scenes (AMOS) project (50), researchers have used historical webcam images to quantify pedestrian/bicyclist use over time at plazas or as street infrastructure changed. Originally a laborious process of manually counting and coding behavior in crowd-sourced photographs (51, 52), AMOS has evolved to now use machine learning to reduce the labor required to code data (53).

Second, is the need to reduce the burden of collecting the data. Systematic observation to assess PA in parks and recreation areas has evolved through the often-used System for Observing Play and Recreation in Communities (SOPARC) (54–57). Initially reliant on in-person observers using hand-written forms, SOPARC has evolved to incorporate iPads (iSOPARC) (58), wearable video detection devices (37), wide-angle cameras mounted on an elevated tripod (36), and remotely controlled aerial vehicles (drones) for data collection (59).

Third is the need to determine the validity of the PIC as a trail usage counter. Although this field test has limitations, our series of analyses suggest a moderate to strong concordance and agreement between the instruments. However, the significant paired *t*-tests suggest there is bias between the infrared sensor and PIC, with the infrared sensor demonstrating higher hourly counts. This bias is shown in the Bland–Altman plots (Figure 4), especially by the wide variance of the mean hourly differences (±10.0) in Figure 4B relative to the overall mean difference (Bias line; 0.82). This shows that for about one third of the observed hours when counts weren't zero, the PIC and sensor differed by 5–10 counts in an hour, with the sensor generally registering higher counts. For example, if a count of 15 was registered by the sensor, the count from the PIC could vary from 5 to 25. Also worth noting in Figure 4 is that the difference between the senor and camera counts (*y*-axis) increased as traffic increased on the trail (*x*-axis). Thus, even though the difference between measures for entire sample was small (mean bias of 0.8), individual-level (hourly) accuracy may need to be improved. This may be due to the detection delay settings of the instruments that may have led to a count of multiple *uses* on the infrared sensor from what would be coded as a single count of one *user* in PIC photos and/or an animal that is only detectable through PIC photos. An interesting finding, albeit exploratory, was the crude calculation of the ICR (percent agreement). We adapted agreement measures from SOPARC studies (36, 37) to fit our data collection methods which are continual over a period of time rather than snapshots of scanned target areas of a larger setting (i.e., park) (54). With limited guidance on the “best practice” of how to establish agreement and concordance among the devices, this study's findings suggest there is a need to establish a method to assess the validity of PIC counts against infrared sensor with concurrent in-person counts in controlled and real-world scenarios, following guidance of similarly purposed SOPARC studies (32, 36, 37). Calculating agreement based on a modified technique often used in qualitative methods demonstrated to be insufficient. Future studies should also consider a cadre of equivalence tests for evaluating measurement agreement between instruments [see (60)].

There are limitations to this study worth noting, including the use of only one type of PIC. Comparisons between camera models and manufacturers will be important to identify the “best performer” for trail use counts; however, this will change as new models become available. Secondly, we used *hourly* counts from the infrared sensor. Timestamp data from the sensor would have provided more precise information about each sensor hit to compare with the camera photos' timestamp, though in-person systematic observation is necessary to determine the true accuracy of both methods. We recommend this timestamp comparison between instruments and in-person observation in any future validation study. The use of the PIC photos, while taken in a public park where trail users anticipate potential observation, also poses potential ethical issues (25, 53). Lastly, camera theft, battery or storage malfunction, and tampering are also a potential concern that may disrupt the research process and increase cost.

## Conclusions

The impact of new or improved trail infrastructure is fertile ground for acquiring insights on PA pattern and usage changes. Dependable, user-friendly, resource-efficient instruments for measuring PA frequency, intensity, time, and type (PA FITT) are critical to influence what is known about the benefits of trail infrastructure improvements. For example, natural experiment studies that explore changes in PA as a response to changes in the built environment often are restricted to self-reported measures of PA which may lack the objectivity and specificity to accurately assess on-trail PA FITT (61–63). This study suggests PICs may be a viable solution to (1) overcome many of these measurement limitations listed above, (2) provide a tool for trail-specific systematic observation of PA FITT, and (3) observe trail usage patterns over time in lieu of resource heavy research designs. Although slightly speculative, PICs may provide opportunities for community engagement by providing virtual public feedback, which may help planners prioritize trail improvement based on actual trail use and behaviors. In addition, PICs may also be applicable to other setting-specific natural experiments such as Open Streets events (64) or temporary street closures such as those enacted during the COVID-19 pandemic (65). Future studies that focus on the reliability of camera settings, installation criteria, and improved analysis of data are critical to establish a best practice for future research.

## Data Availability Statement

The raw data supporting the conclusions of this article will be made available by the authors, without undue reservation.

## Ethics Statement

The protocol was reviewed and approved by the West Virginia University Institutional Review Board (protocol number 1905572292) and deemed Non-Human Subjects Research because it was an observation of public behavior. A written media release was collected from the participants or their legal guardians/next of kin depicted in any images used for public presentation. To protect anonymity, faces were obscured in images selected as figures in this manuscript.

## Author Contributions

CA and VH conceptualized the study, collected and cleaned the data, and drafted the manuscript. SD analyzed the data, and drafted the methods and results sections. TB helped conceptualize and edit the manuscript. All authors have approved the final version and take responsibility for the content.

## Conflict of Interest

The authors declare that the research was conducted in the absence of any commercial or financial relationships that could be construed as a potential conflict of interest.
